# Supplementary material for: Survival of the fittest in the pandemic age: Introducing disease-related social Darwinism
Source: PLoS One. 2023 Mar 22;18(3):e0281072. doi: 10.1371/journal.pone.0281072 (PMC10032491; doi:10.1371/journal.pone.0281072)
Supplement: Attachment — Submitted filename: Response to Reviewers.docx [file pone.0281072.s002.docx]

Dear Dr. Jonason and dear reviewers,

we would like to thank you and the reviewers for the overall positive and constructive feedback on the revised version of our manuscript. We believe that the comments made by Reviewer 1 have helped us to give our manuscript the final polish. Based on these comments and suggestions, we have made further changes to the manuscript, which are detailed in the responses (in italics) below. We think that we were able to address all of the raised concerns. However, if anything remains unsatisfactory, we will happily undertake more revisions. At this point we also want to thank Reviewer 2 for appreciating our revisions after the first round of peer-review and for suggesting our manuscript for publication.

We are looking forward to hearing from you! And thanks again.

Sincerely,

Paul Nachtwey & Eva Walther

Note: the line numbers refer to the unmarked manuscript version, as the line numbers are subject to change in the marked-up version of the document depending on how changes are made visible in Microsoft Word.

**Journal Requirements**

1. Please review your reference list to ensure that it is complete and correct. If you have cited papers that have been retracted, please include the rationale for doing so in the manuscript text, or remove these references and replace them with relevant current references. Any changes to the reference list should be mentioned in the rebuttal letter that accompanies your revised manuscript. If you need to cite a retracted article, indicate the article’s retracted status in the References list and also include a citation and full reference for the retraction notice.

*We appreciate PLOS ONE’s attention to detail. We have checked our reference list and did not find articles that were retracted or needed to be removed. However, while working on the comments made by Reviewer 1, we found a recently published paper investigating social Darwinism during the COVID-19 pandemic, specifically its relationship to ageism (Kanık et al., 2022). Because we think that this paper is relevant to our line of argumentation, we have added a short paragraph in the introduction citing said paper (l. 94-97). Apart from adding this paper to our reference list, no other changes have been made.*

**Reviewer 1**

1. The revised version of the manuscript was substantially improved. The Authors put a great effort in the revision and adressed all suggestions of the reviewers. I have now only a few suggestions:
   There are some possible problems with collinearity presented in Table 4 (DV: protective behavior; IV: Left/Right; DRSD; Interaction). Similar situation is present in Table 7 (see a lot of Tolerance is below .100). I think that the Authors should at least comment on this possible problem and, preferably, explain it.

*First, we want to thank Reviewer 1 for the positive feedback regarding our revisions after the first round of peer-review. Second, we thank Reviewer 1 for bringing up the low Tolerances in some of the regression tables. As can be seen, the low Tolerances in both tables only concern the regression analyses with uncentered predictors. While the tolerances in the regression analyses with centered predictors (marked by © in the tables) are satisfactory. To prevent future confusion, we have added a note to both tables explaining this (see Table 4 &7).*

2. Given the study model which indicates that DRSD is a part/form of social darwinism, I think that the Authors could indicate how these two phenomenons are structurally separate. I think that for the clarity, additional EFA on the items of both DRSD and SD could be beneficial. In example, the Authors could include this analysis only as a note.

*We thank Reviewer 1 for his comments on the relationship of DRSD and SD and suggesting an additional EFA. Because we agree with Reviewer 1, we have calculated the proposed EFA and found that this produces two highly correlated (r = .5) though distinct factors; Factor 1 containing all items of DRSD and Factor 2 all items of SD. This provides support for our conception of DRSD as a structurally distinct part of SD. We have added this analysis in a short paragraph to our manuscript (l. 377-382).*

3. In Study 1 and Study 2, e.g. a Harman's test for detection of common method bias could be an additional information in favor of the Authors standing that the tested variables were indeed independent and the validity analysis was conducted properly.

*We thank Reviewer 1 for bringing up the concern about common method bias. Even though the Harman’s test has been criticized a lot in the literature (e.g., Aguirre-Urreta & Hu, 2019; Baumgartner et al., 2021), we agree with Reviewer 1 that it could provide additional evidence in our favor. Hence, we have calculated the test for both studies and in both studies the single factor extracted explained around 32 % of total variance. This can be interpreted as additional information giving us confidence in our analyses. We have added a short section describing the test for both Studies (l.392-395 & l. 643-646)*

**References**

Aguirre-Urreta, M. I., & Hu, J. (2019). Detecting common method bias: Performance of the Harman’s single-factor test. *ACM SIGMIS Database: The DATABASE for Advances in Information Systems*, *50*(2), 45–70.

Baumgartner, H., Weijters, B., & Pieters, R. (2021). The biasing effect of common method variance: some clarifications. *Journal of the Academy of Marketing Science*, *49*(2), 221–235.

Kanık, B., Uluğ, Ö. M., Solak, N., & Chayinska, M. (2022). “Let the strongest survive”: Ageism and social Darwinism as barriers to supporting policies to benefit older individuals. *Journal of Social Issues*, *78*(4), 790–814.
